# Serum antinuclear antibodies associate with worse prognosis in AQP4‐positive neuromyelitis optica spectrum disorder

**DOI:** 10.1002/brb3.1865

**Published:** 2020-12-14

**Authors:** Rong Fan, Yuefeng Zhang, Yunqi Xu, Jiayi Tong, Zhigang Chen, Meifeng Gu, Wenkui Fan, Yong Chen, Fuhua Peng, Ying Jiang

**Affiliations:** ^1^ Department of Neurology and Multiple Sclerosis Research Center The Third Affiliated Hospital Sun Yat‐sen University Guangzhou China; ^2^ Department of Neurology Guangzhou Brain Hospital Guangzhou China; ^3^ Department of Neurology Nanfang Hospital Southern Medical University Guangzhou China; ^4^ Department of Biostatistics, Epidemiology and Informatics University of Pennsylvania Philadelphia PA USA; ^5^ Department of Neurology The Fifth Affiliated Hospital Sun Yat‐sen University Zhuhai China; ^6^ Department of Nephrology The Second People's Hospital of Foshan Foshan China

**Keywords:** antinuclear antibody, neuromyelitis optica spectrum disorder, prognosis

## Abstract

**Background:**

Studies have demonstrated that antinuclear antibodies (ANAs) may be correlated with neuromyelitis optica spectrum disorder (NMOSD). However, the conflicting results of studies about the value of ANAs in AQP4 antibody‐positive NMOSD patients need to be further investigated.

**Material:**

Case data were collected from 143 patients with AQP4 antibody‐positive NMOSD. Patients were divided into two groups based on the ANA test results. The analysis of clinical characteristics, laboratory tests, and MRI examination results were compared between two groups: the NMOSD patients with ANA (+) and with ANA (−).

**Results:**

Disease duration of NMOSD is shorter in the ANA (+) patients with EDSS < 4 than in the ANA (−) patients (12.05 ± 16.73 versus 29.43 ± 41.03, *p*‐value = .013). The median time from disease onset to an EDSS score of 4.0 is significantly longer in the ANA (−) NMOSD patients than in the ANA (+) patients (48.2 months versus 24 months, *p* = .04). In addition, ANA (RR, 2.234; 95% CI, 1.078–4.629; *p*‐value = .031) can predict the severity of NMOSD.

**Conclusions:**

Antinuclear antibodies seem to be associated with more severe disease activity in NMOSD patients.

## Graphical abstract

ANA+ NMOSD patients with EDSS < 4 have shorter disease duration. The median time from disease onset to EDSS 4.0 is longer in ANA‐ NMOSD patients. ANA can predict the severity of NMOSD.

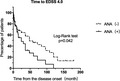

## 1 INTRODUCTION

Neuromyelitis optica spectrum disorder (NMOSD) is an autoimmune disease occurring in the central nervous system (CNS). The term NMOSD was introduced in 2007 (Wingerchuk et al., 2007), and NMOSD is mainly manifested as recurrent optic neuritis (ON) and transverse myelitis (TM), with the clinical diagnostic specificity positive for antibodies to aquaporin‐4 (AQP4) (Wingerchuk et al., 2015). In recent years, increasing evidence has demonstrated that AQP4‐IgG causes damage to peripheral organs beyond the CNS such as skeletal muscle, vestibulocochlear nerves, gastrointestinal tract, blood system, kidney, lung, and placenta (He et al., 2017).

Clinically, a considerable number of NMOSD patients are positive for other autoimmune antibodies including antinuclear antibodies (ANAs), which are produced against DNA, RNA, proteins, or their molecular complexes in the cell nucleus. ANAs can be characterized in many autoimmune diseases such as systemic lupus erythematosus (SLE) and determine the activity and prognosis of SLE (Egner, 2000; Pisetsky, 2017). It has been reported that AQP4‐Ab was detectable in 84.6% patients with SLE and NMO or LETM/rON and in 62.5% patients with other CTD and NMO or LETM, which showed the coexisting relationship between other autoimmune diseases and AQP4‐Ab‐positive NMOSD patients (Jarius et al., 2011). ANAs were reported to be more frequently exist in NMO‐IgG‐seropositive patients (Pittock et al., 2008), while another report also showed that patients with AQP4(+) were more likely to have coexisting autoantibodies (45/97, 46,4%) and autoimmune disorders (31/130, 23.8%) (Jarius et al., 2012). However, it is still unknown whether ANAs have any clinical significance or represent an epiphenomenon in NMOSD. A study showed that ANAs may be a protective marker in NMOSD. In contrast, another study found that ANAs are not sufficient to indicate the severity of NMOSD disease (Lee et al., 2019; Masuda et al., 2016). The contradictory results of these studies motivate us to further investigate the role of ANA in NMOSD.

In this paper, we hypothesized that more severe disease activity may have the potential to be occurred in ANA (+) NMOSD patients, and we conducted to evaluate the value of ANAs in measuring disease severity and prognosis of patients with APQ4 antibody‐positive NMOSD by comparing the clinical characteristics, laboratory tests, and MRI examination results of AQP4 (+) NMOSD patients with ANA (+) or ANA (−).

## 2 METHODS

### 2.1 Samples and study design

This study is approved by the Medical Ethics Committee of the Third Affiliated Hospital of Sun Yat‐sen University (No.2017‐33). All study participants have written informed consent for research and publication.

We recruited 143 AQP4 antibody‐positive NMOSD Chinese Han patients. All the patients were tested for ANAs in the Department of Neurology and Multiple Sclerosis Research Center, the Third Affiliated Hospital of Sun Yat‐sen University, during November 2012 and November 2017. According to the presence of ANAs, the patients were categorized into 52 ANA (+) patients and 91 ANA (−) patients. The diagnosis of NMOSD was based on the 2015 international consensus diagnostic criteria (Wingerchuk et al., 2015). The disability status of NMOSD patients after admission was determined by the use of the Expanded Disability Status Scale (EDSS) before any treatments (Kurtzke, 1983) and classified as mild disability (EDSS score 0–3.5) or moderate/severe disability (EDSS score 4‐9.5) (Conradsson et al., 2018; Sicras‐Mainar et al., 2017). EDSS with score 4.0 (hereafter referred as to EDSS 4.0) is considered to be a key disability milestone (Harding et al., 2019). Thus, EDSS 4.0 was used as the cutoff point in this study. Other systemic autoimmune diseases were diagnosed by rheumatologists according to the established criteria (Aringer et al., 2019; Garber et al., 2012; Ross et al., 2016; Shiboski et al., 2017). The MRI examination time of patients was within 5–7 days after admission at the disease onset period. Clinical characteristics and MRI findings during attacks also were compared between the groups. About 63.5% (33/52) of patients with ANA (+) and 68.1% (62/91) of patients with ANA (−) were first diagnosed by our department, and no immunosuppressive drugs were used before the diagnosis and the ANA tests. The others had received immunosuppressive treatment in other hospitals during the stable period previously. Patients were recruited and tested at the acute phase, and ANA test was performed in the first day morning after admission. After blood samples of ANA test were collected, all NMOSD patients received at least one therapy such as methylprednisolone pulse therapy, intravenous immunoglobulin.

### 2.2 Serological and CSF examination

ANAs were detected with indirect immunofluorescence assay (IIF; EUROIMMUN Medizinische Labordiagnostika AG) in dilution ratios of 1:100, 1:320, 1:1,000, and 1:3,200. Other autoantibodies (anti‐SSA/Ro, anti‐SSB/La, and anti‐TPO) were identified with an ANA profile line immunoblot assay, according to the manufacturer's instructions (HOB Biotech Group) (Chen et al., 2016; Wei et al., 2020). Patients were tested for AQP4 antibody using a cell‐based assay from a commercial BIOCHIP kit (Euroimmun) (Long et al., 2012). Serum samples were diluted to 1:9, and each sample was assayed at least twice, by two examiners blind to the origin of the specimens. Samples with positive results twice were deemed to be positive. We also examined CSF parameters, including white blood cells (WBC) and total protein (TP).

### 2.3 Statistical analyses

All the data in this study were presented as mean ± standard deviation (*SD*) or median ± interquartile range (IQR). Characteristics were compared using Pearson's chi‐square test (or Fisher's exact) for categorical variables and two‐sample *t* test (or nonparametric Mann–Whitney *U* test) for continuous variables. The survival time to an EDSS 4.0 was displayed using the Kaplan–Meier curves; log‐rank test was used to compare the survival experience between the ANA (+) and ANA (−) groups. The prognosis value was analyzed by the Cox multivariate model. Due to the exploratory nature of the study, no adjustment for multiple comparisons was made.

All statistical analyses were performed by the Statistical Program for the Social Sciences (SPSS) statistical software (version 22.0). A two‐sided *p*‐value < .05 was considered statistically significant.

## 3 RESULTS

### 3.1 Demographic and clinical characteristics, laboratory findings in ANA (+) and ANA (−) NMOSD patients

Table 1 shows 86.01% (123/143) of the participants are females, accounting for 88.46% (46/52) in the ANA (+) group and 84.61% (77/91) in the ANA (−) group. The median age is 38.58 ± 11.96 years in the ANA (+) group and 41.08 ± 14.48 years in the ANA (−) group. There are no significant differences in age and sex distribution between ANA (+) patients and ANA (−) patients (*p* > .05). The disease duration in the ANA (+) patients is shorter compared with the ANA (−) patients (18.94 ± 24.48 months versus 30.14 ± 40.20 months, *p* = .044). Autoimmune diseases including Sjogren's syndrome (SS), rheumatoid arthritis (RA), SLE, and hyperthyroidism are confirmed in all of the NMOSD patients, and 28.85% ANA (+) patients are diagnosed with SS, which have a significantly higher incidence than ANA (−) patients (28.85% versus 4.40%, *p* = .001). The main symptoms at onset attack, EDSS, ARR, CSF WBC counts, and CSF TP level are not statistically significant with p‐value larger than 0.05.

**Table 1:** Demographic and clinical characteristics, and laboratory findings in ANA (+) and ANA (−) NMOSD patients

	ANA (+) NMOSD *n* = 52	ANA (−) NMOSD *n* = 91	*p*‐value
Demographic and clinical features			
Gender, male/female	6/46	14/77	.524^b^
Age, years (mean ± *SD*)	38.58 ± 11.96	41.08 ± 14.48	.293
Disease duration, months (mean ± *SD*)	18.94 ± 24.48	30.14 ± 40.20	.044
EDSS, median (IQR)	4.0 (3.0, 5.5)	3.5 (3.0, 5.5)	.221^a^
ARR, median (IQR)	1.48 (0.63, 2.00)	1.00 (0.74, 1.67)	.598^a^
Onset attack, *n* (%)			
ON	24 (24/52, 46.15%)	38 (38/91, 41.76%)	.610^b^
TM	17 (17/52, 32.69%)	35 (35/91, 38.46%)	.490^b^
Both ON and TM	2 (2/52, 3.85%)	7 (7/91, 7.69%)	.362^b^
Others	9 (9/52, 17.31%)	11 (11/91, 12.09%)	.387^b^
Anti‐AQP4‐IgG	52 (52/52, 100.00%)	91 (91/91, 100.00%)	–
Overlapping disorders, *n*(%)			
Sjogren's syndrome	15 (15/52, 28.85%)	1 (1/91, 1.10%)	.001^b^
Rheumatoid arthritis	0 (0/52, 0%)	1 (1/91, 1.10%)	–
Systemic lupus erythematosus	1 (1/52, 1.92%)	0 (0/91, 0%)	–
Hyperthyroidism	4 (4/52, 7.69%)	2 (2/91, 2.20%)	–
CSF findings			
White blood cells, *n*/μl (IQR)	4.0 (0.5, 15.0)	2.0 (0, 10.0)	.118^a^
Total protein, g/L (mean ± *SD*)	0.36 ± 0.27	0.32 ± 0.22	.118^a^

Note

*p* = refers to the comparison between ANA (+) and ANA (−) NMOSD patients.

Abbreviations: ANA, antinuclear antibody; ARR, annualized relapsing rate; CSF, cerebrospinal fluid; EDSS, Kurtzke's Expanded Disability Status Scale; IQR, interquartile range; NMOSD, neuromyelitis optica spectrum disorder; ON, optic neuritis; TM, transverse myelitis.

^a^ Mann–Whitney *U* test.

^b^ Chi‐square test.

### 3.2 Comparison between ANA (+) and ANA (−) NMOSD patients with EDSS score < 4 or EDSS score ≥ 4

The disease duration of NMOSD in the ANA (+) patients is shorter compared with the ANA (−) patients when EDSS score is <4 (12.05 ± 16.73 months versus 29.43 ± 41.03 months, *p* = .013) (Table 2). Statistically significant differences are not found in gender, age, EDSS score, and ARR between ANA (+) and ANA (−) NMOSD patients with EDSS score < 4 or with EDSS score ≥ 4.

**Table 2:** Demographic and clinical characteristics of ANA (+) and ANA (−) NMOSD patients when EDSS < 4 or ≥ 4 score

	EDSS < 4			EDSS ≥ 4		
	ANA (+) NMOSD	ANA (−) NMOSD	*p*‐value	ANA (+) NMOSD	ANA (−) NMOSD	*p*‐value
Patient number	24	49	–	28	42	.376^a^
Gender, male/female	2/22	8/41	.568^a^	4/24	6/36	1.00^a^
Age, years (mean ± *SD*)	39.71 ± 8.61	39.04 ± 12.83	.819	37.61 ± 14.32	43.40 ± 16.00	.119
Disease duration, months (mean ± *SD*)	12.05 ± 16.73	29.43 ± 41.03	.013	24.84 ± 30.15	30.96 ± 39.68	.467
EDSS, median (IQR)	3.0 (2.5, 3.5)	3.0 (2.5, 3.5)	.435^b^	5.5 (5.0, 5.5)	5.5 (5.0, 5.5)	.594^b^
ARR, median (IQR)	1.50 (0.92, 2.00)	1.00 (0.74, 2.00)	.533^b^	1.00 (0.71, 1.46)	1.00 (0.60, 1.81)	.738^b^

Note

*p* refers to the comparison between ANA (+) and ANA (−) NMOSD patients.

Abbreviations: ANA, antinuclear antibody; EDSS, Kurtzke's Expanded Disability Status Scale; NMOSD, neuromyelitis optica spectrum disorder.

^a^ Chi‐square test.

^b^ Mann–Whitney *U* test.

**FIGURE 1:**
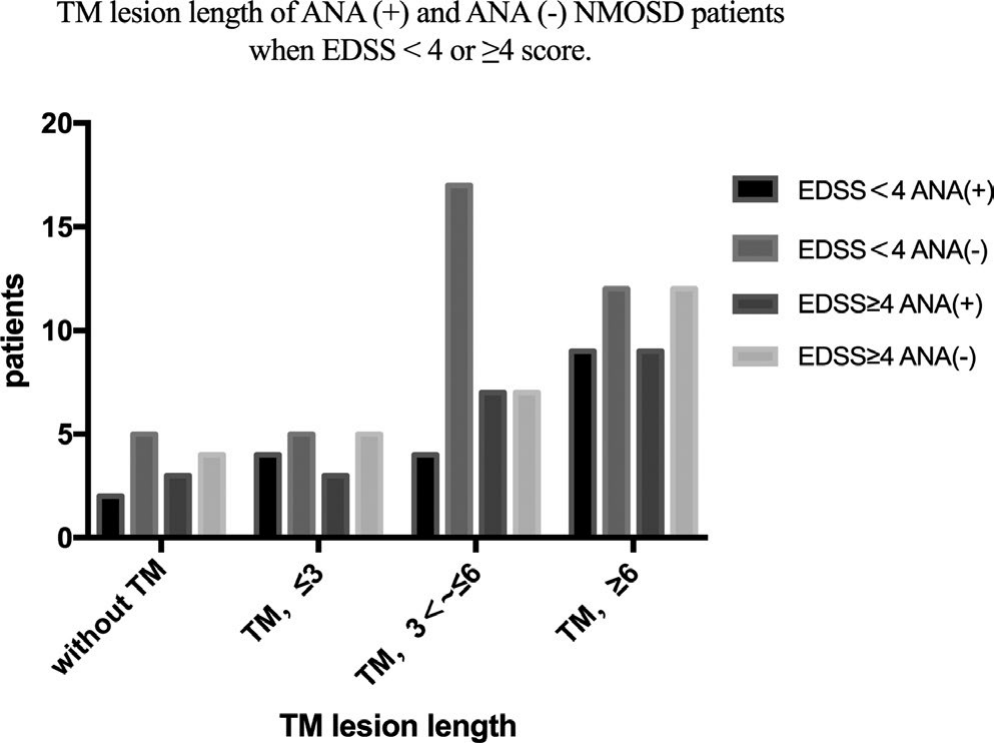
TM lesion length of EDSS score < 4 and EDSS score ≥ 4 in ANA (+) and ANA (−) NMOSD patients

The number of patients with longitudinal extensive TM in MRI (≥3 segments or more) is much higher, especially in the ANA (−) group (Figure 1). 17 (17/39, 43.59%) ANA (−) patients have the length of TM lesion from 3 to 6 segments when EDSS < 4, which is more than ANA (+) patients, but without statistical significance (17/39, 43.59% versus 4/19, 21.05%, *p* = .094) (Table 3).

**Table 3:** Comparisons about TM lesion lengths between ANA (+) and ANA (−) NMOSD patients when EDSS < 4 or ≥ 4 score

	EDSS < 4 *n* = 58			EDSS ≥ 4 *n* = 50		
	ANA (+) NMOSD	ANA (−) NMOSD	*p*‐value	ANA (+) NMOSD	ANA (−) NMOSD	*p*‐value
TM lesion length^c^			.345^b^			.950^b^
Without TM	2 (2/19, 10.53%)	5 (5/39, 12.82%)	.803^a^	3 (3/22, 13.64%)	4 (4/28, 14.29%)	.948^a^
TM, <3	4 (4/19, 21.05%)	5 (5/39, 12.82%)	.420^a^	3 (3/22, 13.64%)	5 (5/28, 17.86%)	.689^a^
TM, 3≤ ~ <6	4 (4/19, 21.05%)	17 (17/39, 43.59%)	.094^a^	7 (7/22, 31.82%)	7 (7/28, 25.00%)	.594^a^
TM, ≥6	9 (9/19, 47.37%)	12 (12/39, 30.77%)	.217^a^	9 (9/22, 40.91%)	12 (12/28, 42.86%)	.890^a^

Note

*p* refers to the comparison between ANA (+) and ANA (−) NMOSD patients.

Abbreviations: ANA, antinuclear antibody; EDSS, Kurtzke's Expanded Disability Status Scale; NMOSD, neuromyelitis optica spectrum disorder; TM, transverse myelitis.

^a^ Chi‐square test

^b^ Kruskal–Wallis test was used for the comparison among 4 groups of TM lesion length.

^c^ A total of 108 patients underwent MRI examination, including 41 patients with ANA (+) NMOSD and 67 patients with ANA (−) NMOSD.

### 3.3 Comparison of time to an EDSS score of 4.0 between ANA (+) and ANA (−) NMOSD patients

**FIGURE 2:**
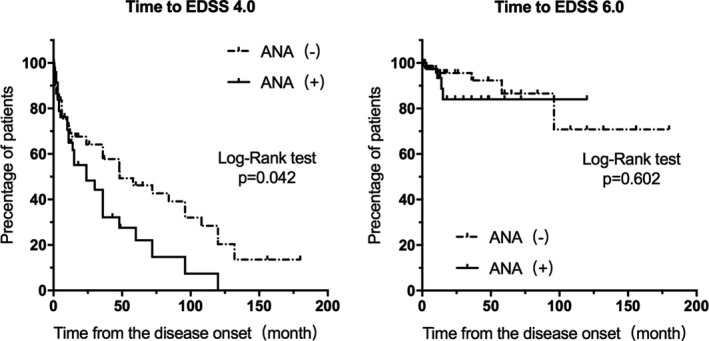
Kaplan–Meier survival curves of time from the onset of NMOSD to an EDSS score of 4.0 or EDSS score of 6.0 in ANA (+) NMOSD patients (solid line) and ANA (−) NMOSD patients (dashed line). ANA, antinuclear antibody; EDSS, Kurtzke's Expanded Disability Status Scale; NMOSD, neuromyelitis optica spectrum disorder

The median time from disease onset to an EDSS score of 4.0 is significantly longer in the ANA (−) NMOSD patients compared with the ANA (+) patients (48.2 months versus 24 months, *p* = .04). The results with the Kaplan–Meier analysis reveal the significant difference between the ANA groups in the EDSS 4.0 achievement rate, but reveal no significant difference between the ANA groups in the EDSS 6.0 achievement rate (*p* = .602) (Figure 2). Multivariate Cox proportional hazards regression analysis is used to evaluate the clinical value for ANAs as significant predictors for the disease severity, which indicates that ANAs (RR, 2.234; 95% CI, 1.078–4.629; *p* = .031) and ARR (RR, 3.845; 95% CI, 2.1573–6.852; *p* < .001) could predict the severity of NMOSD.

## 4 DISCUSSION

With the deepening research on NMOSD and its related AQP4 autoantibody, more attention has been attracted on the relation between NMOSD and other autoimmune antibodies such as ANAs. A few studies have been conducted to investigate the value of ANAs in evaluating disease severity and prognosis of NMOSD patients. However, the conflicting results of the studies about the value of ANAs in NMOSD patients need to be further investigated. In this paper, we conducted clinical characteristics, laboratory tests, and MRI findings between the AQP4 antibody‐positive NMOSD patients with and without ANA autoantibodies. With statistical tests and Cox proportional hazards model, we found that the disease duration of NMOSD is shorter in the ANA (+) patients with EDSS < 4.0 when compared to the ANA (−) patients. The median time from disease onset to an EDSS score of 4.0 is significantly longer in the ANA (−) NMOSD patients when compared to the ANA (+) patients. In addition, ANA can be used to predict the severity of NMOSD.

NMOSD can coexist with other autoimmune diseases, including SLE, SS, and autoimmune thyroiditis (Lana‐Peixoto, 2008; Zekeridou & Lennon, 2015). Serum ANAs also remain the most used diagnostic biomarkers of these autoimmune diseases, which are targeted with the nuclear membrane, chromatin, nonhistone proteins, ribonucleic acid, and RNA‐associated proteins as the most common (Goulvestre, 2006). The NMOSD patients can be ANA‐positive with a frequency range between 31% and 82.6% (Long et al., 2013; Wu et al., 2014; Pereira et al., 2017; Gkaniatsou et al., 2020), generally with lower titers. The most common range of ANA‐positive frequency is around 40%, which is similar to the results of the study we conducted (36.4%, 52/143). The frequency of ANAs in NMOSD and the significance remains a matter of debate. In most patients (42/52, 80.77%) with an ANA titer of 1:100, this level of ANA is not known to have pathogenic significance in NMOSD, but the occurrence of ANA may reflect the systemic immune dysregulation that may be ongoing in NMOSD. Sex is one of the top risk factors in NMOSD, with women being more often affected than men. Sex ratio heavily depends on the antibody status, while the female‐to‐male ratio was reported to be 9–10:1 in seropositive patients (Gold et al., 2019). Similar result was found in this study that 86.01% (123/143) of the participants are females, accounting for 88.46% (46/52) in the ANA (+) group and 84.61% (77/91) in the ANA (−) group. Age was considered to be associated with autoreactive antibodies (Ruffati et al., 1990). However, the mean age did not differ between ANA‐positive and ANA‐negative NMOSD patients, or between NMOSD patients with and without high levels of ANA. In some patients with symptoms of NMOSD, no AQP4‐Abs but Abs against myelin‐oligodendrocyte‐glycoprotein (MOG) are detectable. These clinical syndromes are now frequently referred to as “MOG‐encephalomyelitis” (MOG‐EM). Although the frequency of coexistent autoimmune diseases seems to be lower than AQP4‐Ab‐positive patients, comorbidity with other autoimmune disorders has been reported in MOG‐EM patients (Borisow et al., 2018). Since all patients in this study were recruited as AQP4 antibody‐positive NMOSD Chinese Han patients, the relationship between ANAs and MOG‐EM is worth to follow in the future. Steroid‐treated patients appeared to have a much lower frequency of ANA than non‐steroid‐treated patients (Pozzilli et al., 1998). The ANA test in this study was performed at the time of the patient's first visit and before the use of steroid treatment. This time point suggests that steroids may have little effect on ANA status in this study.

In this study, the clinical characteristics including the main symptoms of the onset attack, EDSS score, ARR, and CSF examinations do not differ between the ANA groups, which are consistent with previous study (Lee et al., 2019). Besides, we found the time from the onset of NMOSD to moderate disability level with EDSS 4.0 was shorter in the ANA (+) patients than in the ANA (−) patients, indicating the associative relation between ANAs and worse NMOSD prognosis. A latest study found that there were no significant differences between 32 ANA (+) NMOSD patients and 42 ANA (−) NMOSD patients in the EDSS 6.0 achievement rate (Lee et al., 2019). In addition, another study among 19 ANA (+) NMO patients and 44 ANA (−) NMO patients found that ANA (+) NMO patients had a lower ARR, and the time to reach EDSS score of 6.0 was shorter than that with negative ANAs (Masuda et al., 2016). With the EDSS score of 6.0 as the cutoff, we conducted the sensitivity analysis. We analyzed the time to the EDSS score of 6.0 using the Kaplan–Meier curves, but no significant difference was found. The number of patients in the above studies was smaller than ours and relied on EDSS score of 6.0 as the determination of disease severity, leading to the differences in results from what we got. The presence of ANA reflects ongoing autoimmune stimulation by the exposition of self‐antigens during oligodendrocyte apoptosis, which is associated with certain pathological patterns of MS. Early apoptotic oligodendrocyte lesion has been postulated as an early event (Barnett & Sutton, 2006), and ANAs were detected mainly in patients with early diseases (Szmyrka‐Kaczmarek et al., 2012), which means EDSS score of 4.0 is more commonly used as the reference of early and middle stage of inflammatory demyelinating disease of the human central nervous system. Therefore, in order to study the clinical predictive value of ANAs in NMOSD more accurately, EDSS 4.0 is reasonable to be selected as the cutoff point to evaluate the severity and prognosis of the disease.

According to the results we got, ANAs were observed to be associated with the poorer NMOSD prognosis. Another study showed the similar results that primary and secondary progressive disease had a higher antibody frequency than relapsing–remitting (*p* < .05) or benign (*p* < .001) MS (Spadaro et al., 1999). Potential mechanisms have been hypothesized that a generalized immune dysregulation occurred, involving activation of both autoreactive Th1 cells (mainly linked to CNS lesions) and B cells via Th2 cells (Cerutti et al., 2005; Desai et al., 1993). Besides, ANAs could cause inflammation and tissue damage through binding to exposed chromatin fragments or directly to intrinsic antigens by cross‐reactivity, or deposing in tissue lesions by forming immune complexes of antibodies with DNA or nucleosomes (Fenton et al., 2009; Rekvig, 2015; Seredkina et al., 2013).

### 4.1 Limitations

With the advantages of large simple size and prognostic analysis of NMOSD patients with ANA (+), there still exist a few limitations of our study. The major one is that the cross‐sectional studies do not allow inferences on causal relationships. To conduct a longitudinal study on the changes of ANAs in NMOSD patients could help us better understand the causal relationship in the future. Besides, further exploration of the relationship between different titers of ANAs and NMOSD disease is also needed for figuring out the effect of ANA antibody titer on the development of disease. In addition, it is true that there are some patients who had received immunosuppressive treatment or IVIG treatment in other hospitals during the stable period previously, and the results of ANA test were very hard to be collected at the time of their initial onset. Therefore, we did not conduct long‐term follow‐up on ANA status, which is also a limitation of this study. Further study is needed about the relationship among different treatments, ANA status changes, and EDSS scores.

## 5 CONCLUSIONS

In conclusion, our results suggested that ANA seems to be more associated with the severe disease activity changes in AQP4 antibody‐positive NMOSD patients, which further implied that ANA can be able to use as a prognostic marker in NMOSD.

## CONFLICT OF INTEREST

All authors declare that there are no conflicts of interest.

## AUTHORS' CONTRIBUTIONS

Y. J. and F. P. contributed to the conception and design of this study. R. F., Y. Z., Y. X., Z. C., M. G., and W. F. collected and organized the data. R. F., Y. Z., Y. X., J. T., Y. C., F. P., and Y.J. analyzed the data. R. F., Y. Z., Y. X., J. T., Y. C., F. P., and Y.J. drafted the manuscript. All the authors read and approved the final manuscript.

## ETHICAL APPROVAL

The study was conducted according to the principles expressed in the Declaration of Helsinki and approved by the Medical Ethics Committee of the Third Affiliated Hospital of Sun Yat‐sen University (No.2017–33). All study participants gave written informed consent for research and publication.

### Peer Review

The peer review history for this article is available at https://publons.com/publon/10.1002/brb3.1865.

## Data Availability

The datasets used and/or analyzed during the current study are available from the corresponding author upon reasonable request.
